# Early Pseudoprogression Mimicking Pneumonitis After Tarlatamab Therapy: A Case Suggestive of Immune Cell‐Associated Respiratory Syndrome (ICARS)

**DOI:** 10.1002/rcr2.70425

**Published:** 2025-12-01

**Authors:** Natsumi Kushima, Toyoshi Yanagihara, Takato Ikeda, Noriyuki Ebi, Hiroyuki Inoue, Masaki Fujita

**Affiliations:** ^1^ Department of Respiratory Medicine Fukuoka University Hospital Fukuoka Japan

**Keywords:** cytokine release syndrome, immune cell‐associated respiratory syndrome, pseudoprogression, small‐cell lung cancer, tarlatamab

## Abstract

Tarlatamab, a DLL3‐targeting bispecific T‐cell engager, is an emerging option for previously treated small‐cell lung cancer (SCLC). Pseudoprogression with tarlatamab is poorly defined and can mimic drug‐induced pneumonitis. A 77‐year‐old man with extensive‐stage SCLC developed fever and hypoxia by Day 3 after the first 1‐mg dose, with rapid tumour enlargement and diffuse left‐lung infiltrates. High‐dose methylprednisolone and tocilizumab produced prompt clinical and radiographic improvement; follow‐up imaging showed resolution of infiltrates and tumour regression below baseline, consistent with pseudoprogression. This case suggests a reversible, lung‐predominant immune reaction with acute peritumoral infiltration likely driven by T cells. We propose the provisional term ‘Immune Cell‐Associated Respiratory Syndrome (ICARS)’. We speculate that baseline carcinomatous lymphangitis may predispose to ICARS. Recognising ICARS may prevent misdiagnosis and avoid premature discontinuation of effective therapy.

## Graphical abstract

*Tarlatamab* can trigger early, lung‐dominant pseudoprogression characterized by acute, likely T‐cell–driven peritumoral infiltration and transient diffuse infiltrates. We propose the provisional term Immune Cell‐Associated Respiratory Syndrome (ICARS) for this reversible respiratory manifestation, which helps avoid premature discontinuation of effective therapy.

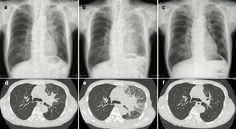

## 1 Introduction

Small‐cell lung cancer (SCLC) is an aggressive malignancy with a poor prognosis, particularly after multiple lines of therapy. Tarlatamab, a bispecific T‐cell engager (BiTE) antibody that redirects T‐cells to target delta‐like ligand 3 (DLL3) on SCLC cells, has shown promising efficacy in the previously treated patients with SCLC [1].

Pseudoprogression, a transient radiographic increase in tumour burden on imaging due to immune cell infiltration, is well‐recognised with immune checkpoint inhibitors (ICIs) [2]. Although considered rare with tarlatamab, two recent case reports have described pseudoprogression: one involving thoracic and hepatic lesions [3], and another involving brain metastases. Similar immune‐driven ‘tumour flare’ phenomena have also been observed with other BiTE therapies, including epcoritamab in lymphoma and talquetamab or teclistamab in multiple myeloma, suggesting a potential class effect of T‐cell‐redirecting agents [4].

Here, we report a case of acute, lung‐dominant pseudoprogression following tarlatamab therapy, characterised by fever, hypoxia, and transient diffuse infiltrates. Based on the clinical features, we propose the provisional concept of ‘Immune Cell‐Associated Respiratory Syndrome (ICARS)’ to describe this reversible respiratory manifestation most likely driven by T cells.

## 2 Case Report

A 77‐year‐old man was diagnosed with extensive‐stage SCLC (cT4N3M1a, cStage IVA) 2 years prior. His medical history included multiple cerebral infarctions and chronic kidney disease. He was an ex‐smoker with a 55 pack‐year history.

**FIGURE 1:**
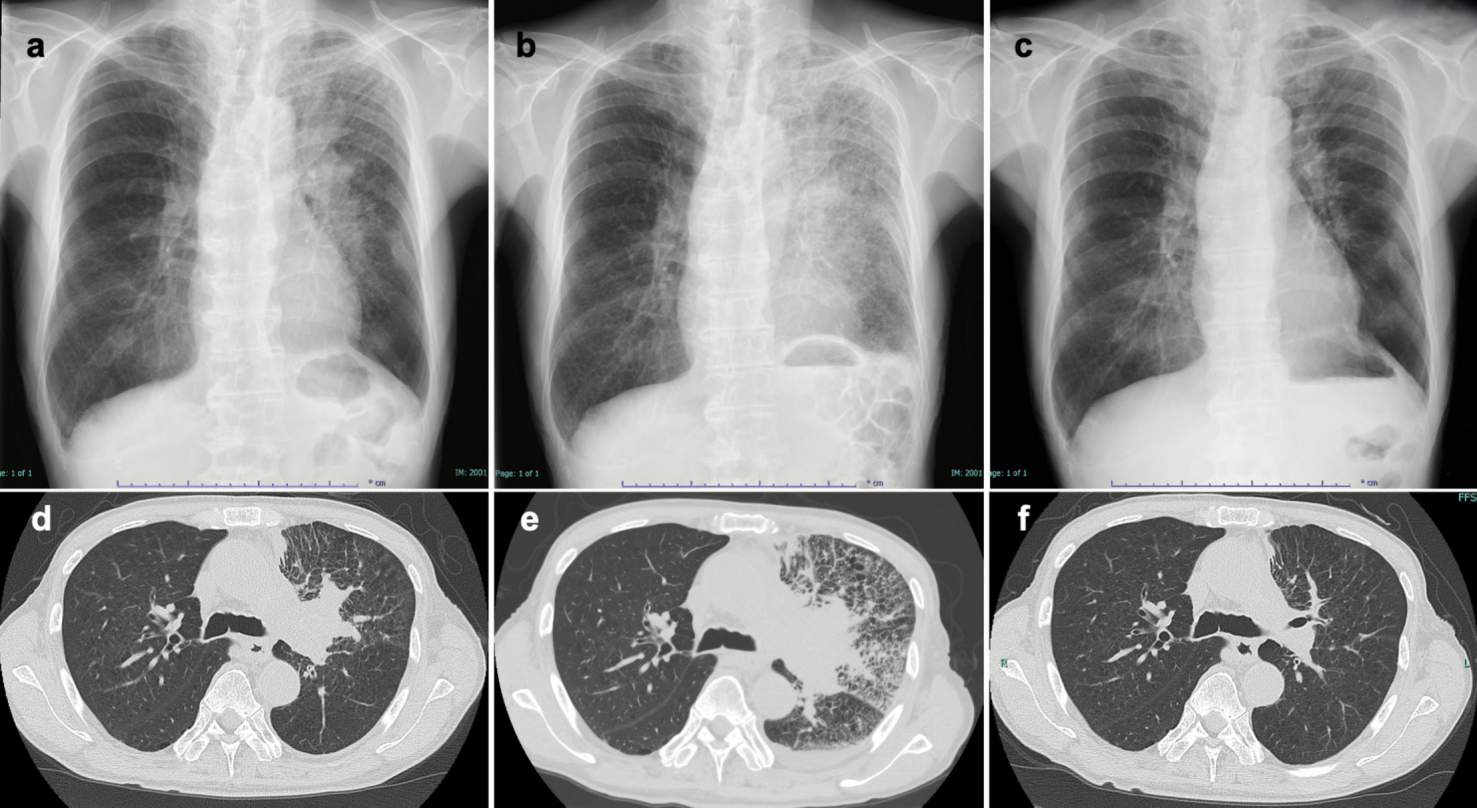
Serial chest imaging before and after tarlatamab administration. (A) Baseline chest x‐ray showing a mass in the left upper lung field; (B) chest x‐ray on Day 3 showing extensive ground‐glass opacities and infiltrates in the left lung; (C) chest x‐ray on Day 28 after the first dose showing resolution of infiltrates and a left hilar mass smaller than at baseline. Chest CT images obtained at baseline (D), on Day 3 (E), and on Day 38 (F).

He had received five prior lines of therapy: (1) carboplatin + etoposide (1 cycle), followed by carboplatin + etoposide + atezolizumab (3 cycles) with atezolizumab maintenance until progression; (2) Amrubicin; (3) Nogitecan; (4) carboplatin + etoposide (re‐challenge); and (5) nab‐paclitaxel, which was discontinued due to disease progression with suspected carcinomatous lymphangitis. Sixth‐line therapy with tarlatamab was initiated. At baseline, his Eastern Cooperative Oncology Group (ECOG) performance status was 1. Baseline chest x‐ray and CT showed a left hilar mass with diffuse reticular opacities suggestive of lymphangitis (Figure 1A,D).

The first 1‐mg dose of tarlatamab was administered on Day 1. On Day 2, he developed a fever of 38°C, which persisted despite acetaminophen. On Day 3, his fever rose to 38.7°C, with tachycardia (110 bpm) and hypoxia (SpO_2_ 93% on room air). Chest x‐ray (Figure 1B) and CT (Figure 1E) on Day 3 demonstrated rapid enlargement of the primary tumour and diffuse infiltrates throughout the left lung, obscuring the mass. Serum CRP level was elevated at 16.27 mg/dL. These findings raised concern for grade 2 cytokine release syndrome (CRS) and/or drug‐induced pneumonitis.

The patient was immediately treated with tocilizumab (8 mg/kg) and high‐dose methylprednisolone (125 mg daily for 3 days), followed by a tapering course of oral prednisolone. His fever and hypoxia resolved promptly. Serial imaging showed rapid improvement: A chest x‐ray on day 14 demonstrated near‐complete clearing of the pulmonary infiltrates. Given the clinical stability and radiographic resolution, this episode was judged to represent pseudoprogression rather than true pneumonitis. Tarlatamab was successfully resumed at a 10‐mg dose on Day 15. A follow‐up chest x‐ray on Day 28 (Figure 1C) and chest CT images on Day 38 confirmed further resolution of infiltrates and reduction of the primary tumour to smaller than baseline.

## 3 Discussion

This case suggests that tarlatamab can cause acute, severe pseudoprogression within days of the first dose and that it closely mimics drug‐induced pneumonitis or rapid progression. On Day 3, tumour enlargement, diffuse infiltrates, and hypoxia were indistinguishable from these life‐threatening conditions. Three findings favoured pseudoprogression: very rapid onset (Days 2–3, atypical for classic pneumonitis); peritumoral/lymphangitic predominance of infiltrates; and prompt, complete resolution after tocilizumab and corticosteroids, followed by tumour regression below baseline.

The mechanism aligns with BiTE pharmacology: tarlatamab redirects T cells to DLL3‐positive tumour cells, causing strong T‐cell activation and cytokine release. The radiographic flare coincided with Grade 2 CRS (fever, hypoxia), supporting a localised, T‐cell–driven inflammatory reaction. Baseline lymphangitic carcinomatosis likely provided a broad antigenic field for redirected T‐cell engagement, enabling diffuse infiltration along tumour‐laden lymphatic channels and producing a lung‐dominant but reversible response.

Tarlatamab has also been associated with Immune Effector Cell–Associated Neurotoxicity Syndrome (ICANS)—a neuroinflammatory complication that often occurs concurrently or sequentially with CRS [5]. Both ICANS and the respiratory event observed here likely represent organ‐specific manifestations of immune effector cell activation. While ICANS reflects T‐cell–mediated neuroinflammation affecting the central nervous system, the current case illustrates a lung‐dominant counterpart characterised by peritumoral T‐cell infiltration and transient respiratory dysfunction. We therefore propose that this presentation represents a distinct, lung‐dominant manifestation of immune effector cell activation, for which we suggest the provisional term ‘ICARS’. ICARS describes a transient, T‐cell–mediated respiratory reaction overlapping clinically with CRS but radiographically dominated by peritumoral and parenchymal lung involvement.

Early anti‐inflammatory treatment led to full recovery and safe reinitiation of tarlatamab, confirming pseudoprogression. As use of T‐cell–redirecting agents expands, recognising ICARS can prevent misdiagnosis and avoid premature discontinuation of effective therapy.

## Author Contributions

Natsumi Kushima and Toyoshi Yanagihara conceived the study, prepared the data and drafted the manuscript. Takato Ikeda, Noriyuki Ebi, and Hiroyuki Inoue interpreted the data and revised the manuscript. Masaki Fujita supervised the study and reviewed the manuscript. All authors approved the final version of the manuscript.

## Funding

The authors have nothing to report.

## Consent

The authors declare that written informed consent was obtained for the publication of this manuscript and accompanying images using the consent form provided by the Journal.

## Conflicts of Interest

The authors declare no conflicts of interest.

## Data Availability

The data that support the findings of this study are available on request from the corresponding author. The data are not publicly available due to privacy or ethical restrictions.
